# Differential effects of genistein and 8-prenylgenistein on reproductive tissues in immature female mice

**DOI:** 10.1080/13880209.2019.1590422

**Published:** 2019-04-04

**Authors:** Xiao-Li Li, Li Sui, Fu-Hui Lin, Yin Lian, Lian-Zhong Ai, Yan Zhang

**Affiliations:** a School of Medical Instrument and Food Engineering, University of Shanghai for Science and Technology, Shanghai, P. R. China;; b Department of Orthopaedic, Shenzhen Pingle Orthopaedic Hospital, Shenzhen, P. R. China;; c Spine Disease Research Institute, Longhua Hospital, Affiliated to Shanghai University of Traditional Chinese Medicine, Shanghai, P. R. China;; d Key Laboratory of Theory and Therapy of Muscles and Bones, Ministry of Education, Shanghai, P. R. China

**Keywords:** Oestrogen receptor, phytoestrogen, uterus, vagina

## Abstract

**Context:** We identified an active prenylated derivative of genistein, 8-prenylgenistein (8PG) from *Erythrina variegata* L. (Leguminosae) and found that 8PG increased osteoprotective effects of genistein in oestrogen-deficient mice.

**Objective:** This study investigated and compared the oestrogenic effects of genistein and 8PG on uterus and vagina of immature mice.

**Materials and methods:** Immature female CD-1 mice were orally treated with vehicle (Control, *n* = 10) or genistein (75 mg/kg, *n* = 10) or 8PG with low (8PG-L, 75 mg/kg, *n* = 10) and high dose (8PG-H, 150 mg/kg, *n* = 10) for 7 consecutive days by intragastric gavage. The uterus and vagina were harvested for histological and molecular measurements.

**Results:** Treatment with genistein and 8PG-H significantly increased uterus index (1.98 ± 0.21 & 1.49 ± 0.16 mg/g) and vagina index (3.83 ± 0.11 & 3.13 ± 0.25 mg/g) as compared to untreated control (uterus, 1.12 ± 0.13 mg/g; vagina, 2.32 ± 0.18 mg/g). Accordingly, both genistein and 8PG-H made vaginal cells keratinized and induced uterine and vaginal hypertrophy associated with the endometrial proliferation. 8PG-L did not affect oestrus cycle and histology of uterus and vagina. Treatment of immature mice with genistein or 8PG-H upregulated protein expression of oestrogen receptor-α (ER-α) and proliferating cell nuclear antigen (PCNA), but 8PG-L did not alter ER-α and PCNA expression in uterus and vagina.

**Conclusion:** This study indicated that 8-prenylgenistein exerted oestrogenic effects in immature female mice. The efficacy and safety of 8-prenylgenistein when applied in improving oestrogen deficiency-induced syndrome requires further elucidation.

## Introduction

Prenylated flavanones are a unique class of naturally occurring flavonoids characterized by the presence of a prenylated side chain in the flavonoid skeleton (Štulíková et al. 2018). The research on prenylated flavanones is a topic of great interest due to their promising and diverse bioactivities on multi-targeting tissues (Štulíková et al. 2018; Zhu et al. 2018). Intriguingly, the structure–activity relationship studies demonstrated that the prenylation at site 8 of ring A in flavonoid structure could strengthen the activities of the original compound as well as potentially produce more effects (Chen et al. 2014). The prenyl group contributed to oestrogenic activity of phytoestrogen 8-prenynaringenin (Štulíková et al. 2018; Hoffmann et al. 2016) which showed stronger bone-protective effects than that of naringenin (Ming et al. 2013).

*Erythrina variegata* L. (Leguminosae) (EV), distributed in China, India, and Southeast Asia and of the same leguminous family as soybean, is used to treat rheumatic joint pain, spasm of the limbs as well as lower back and knee pain, and to stimulate lactation and menstruation for women (Zhang et al. 2007). We previously reported that EV exhibited osteopreserve effects in ovariectomized rats (Zhang et al. 2007) and mice (Zhang et al. 2010) and identified types of prenylated isoflavones with soy isoflavone genistein as the backbone from EV (Li et al. 2006). Among these prenylated genistein derivatives, using *in vitro* biological measurements, we revealed that 8-prenylgenistein (8PG) could increase the ability in stimulating osteoblastic function in comparison to genistein (Zhang et al. 2008). Additionally, the phytoestrogen-free diet supplemented with 8PG (300 ppm) protected against oestrogen deficiency-induced osteoporosis in mice and 8PG (300 & 600 ppm) did not produce the uterotrophic effect that was shown in genistein-treated ovariectomized mice (Zhang et al. 2018).

The present study investigates the effects of 8PG on oestrogen-targeting tissues, such as uterus and vagina, in immature female mice after short-term oral administration and evaluates the *in vivo* safety and the oestrogenic activity of 8PG.

## Materials and methods

### Animals and treatments

A total of 40 one-month-old immature female CD-1 mice (Slac Laboratory, Shanghai, China) were housed in environmentally controlled central animal facilities with alternating 12 h periods of light and darkness, a constant temperature of 23 ± 1 °C, and humidity of 55 ± 5% upon arrival. The mice were allowed free access to deionized distilled water and phytoestrogen-free diet (D00031602, Research Diet Inc, New Brunswick, NJ). The mice were orally treated with vehicle (*n* = 10, Control), genistein (75 mg/kg, *n* = 10), low dose (75 mg/kg, *n* = 10, 8PG-L) or high dose (150 mg/kg, *n* = 10, 8PG-H) of 8-prenylgenistein (8PG) by intragastric gavage. The doses used for genistein and 8PG were referred as described previously (Zhang et al. 2018). Genistein with purity more than 98% was purchased from Sigma and 8PG with purity more than 99% was synthesized in Changzhou University (China). After treatment for 7 days, the uterus and vagina were aseptically removed, weighed, and stored at −80 °C for molecular analysis. Tissue index for uterus and vagina was calculated and expressed as the ratio (mg/g) of tissue weight and body weight. The research was conducted in accordance with the US guidelines (NIH publication #85-23, revised in 1985). All procedures were reviewed and approved by the Animal Ethics Committee of University of Shanghai for Science and Technology.

### Monitoring oestrus cycle

All mice were monitored by daily vaginal epithelium cells smear testing during the 7-day administration period. The vaginal lavage was fixed with 95% ethanol for 10 min and stained with methylene blue for 10 min. The vaginal epithelial cells were observed by microscopy and keratinized vaginal cells were taken as being indicative of oestrogenic activity.

### Histological staining on uterus and vagina

The uterus and vagina were fixed with 4% formaldehyde in phosphate-buffered saline (pH 7.2), processed, embedded in paraffin, and cut into 3 μm sections. The paraffin sections of uterus and vagina were stained with haematoxylin and eosin (HE) and the images were capture using microscopy.

### Immunoblotting

The proteins were extracted in Laemmli buffer (Boston Bioproducts, Worcester, MA). Protein samples were separated on SDS-PAGE gel, transferred to PVDF membranes (Whatman). After saturation with 5% (w/v) non-fat dry milk in TBS and 0.1% (w/v) Tween 20 (TBST), the membranes were incubated with one of the following primary antibodies (abcam), rabbit anti-oestrogen receptor-α (ER-α) and anti-oestrogen receptor-β (ER-β) polyclonal antibodies, and mouse anti-proliferating cell nuclear antigen (PCNA) monoclonal antibody. After washes with TBST, the membranes were incubated with second antibody conjugated with horseradish peroxidase (Beyotime, China). The antigen–antibody complexes were then detected with enhanced chemiluminescence reagent and visualized by the Lumi-Imager using Lumi-Analyst version 3.10 software (Roche, Mannheim, Germany).

### Statistical analysis

The data from these experiments were reported as mean ± standard error of mean (SEM) for each group. All statistical analyses were performed using PRISM version 4.0 (GraphPad). Inter-group differences were analysed by one-way ANOVA and followed by Tukey’s multiple comparison test as a post-test to compare the group means if overall *p* < 0.05. Differences with *p* < 0.05 were considered statistically significant.

## Results

### Effect of 8-prenylgenistein on oestrus cycle

**Figure 1.**
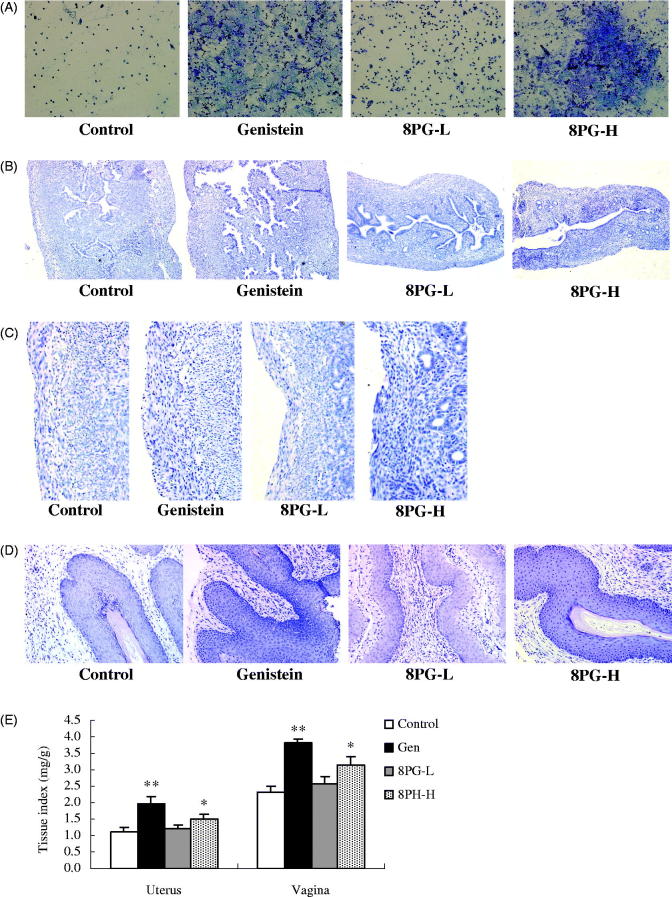
Oestrous cycle, histology of uterus and vagina and tissue index of immature mice treated with vehicle (Control), genistein (Gen) or low (8PG-L) or high (8PG-H) dose of 8-prenylgenistein for 7 days. (A) oestrous cycle (200×). (B) histology of uterus (100×). (C) the uterine epithelium (100×). (D) histology of vagina (200×). (E) uterus and vagina index expressed as the ratio (tissue index, mg/g) of tissue weight and body weight. Values were expressed as means ± SEM, *n* = 10. *p < 0.05, **p < 0.01, vs. Control group.

The influence of 8PG on oestrus cycle in comparison with genistein was studied to characterize the oestrogenic activity of 8PG (Figure 1(A)). The oestrus cycle of immature mice were daily monitored for vaginal epithelial cell smears. The dioestrus smear showed the presence of leukocytes in untreated and 8PG-L-treated immature mice. In contrast, the vaginal epithelial cells from immature mice treated with genistein or 8PG-H became keratinized.

### Effect of 8-prenylgenistein on histology of uterus and vagina

Histological analysis on uterine sections showed that treatment of immature mice with genistein and high dose of 8PG substantially changed uterine morphology as demonstrated by increased number of glands, more extended glandular cavities (Figure 1(B)) and thickening of the uterine endometrium (Figure 1(C)) compared with that of untreated control. Similarly, the representative image of vagina from genistein-treated group displayed a typical squamous multilayered epithelial cell layers with cornification (Figure 1(D)). Treatment with high dose of 8PG enhanced epithelial thickness and the number of cell layers in vagina of immature mice. However, low dose of 8PG did not affect the histology of uterus and vagina of immature mice.

### Effect of 8-prenylgenistein on weight of uterus and vagina

In accordance with the findings on histology of uterus and vagina, genistein and high dose of 8PG significantly increased uterus index and vagina index of immature mice as compared to those of untreated immature mice (Figure 1(E)). However, the indexes of uterus and vagina were not changed in immature mice treated with 8PG-L.

### Effect of 8-prenylgenistein on protein expression of ER-α, ER-β and PCNA

**Figure 2.**
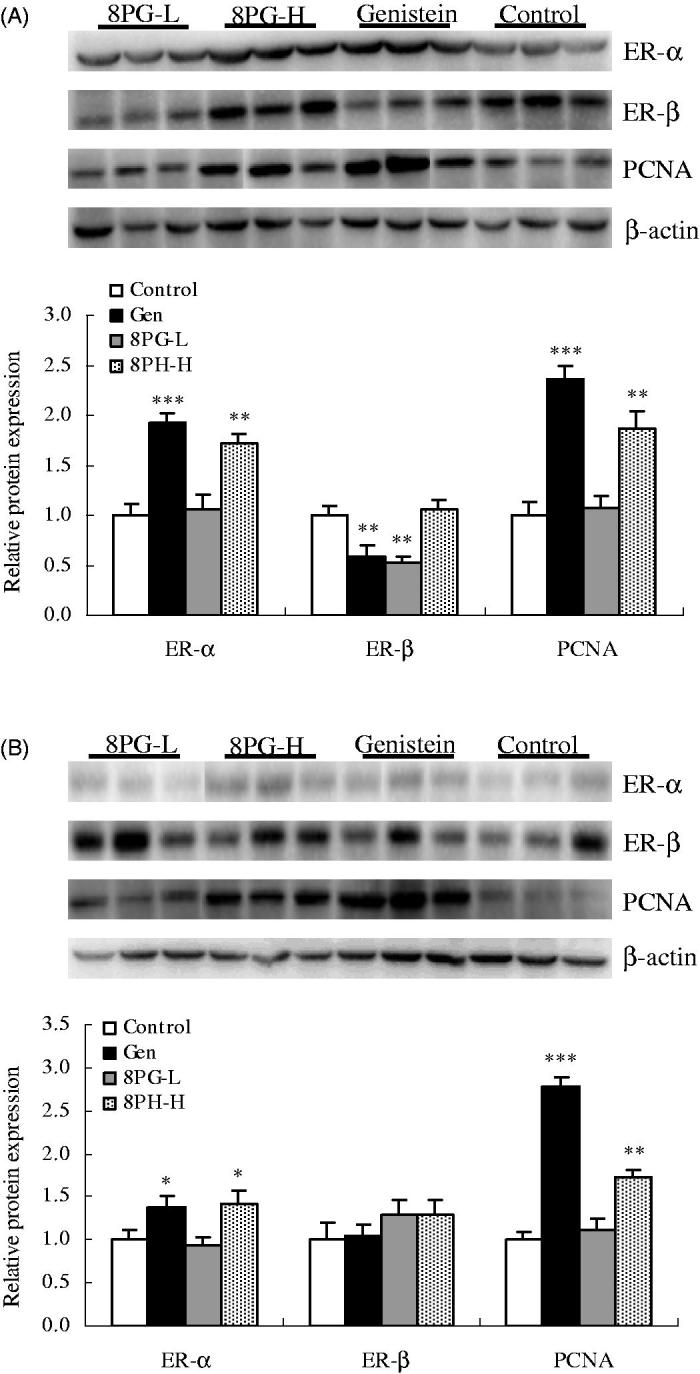
Protein expression of oestrogen receptor-alpha (ER-α), oestrogen receptor-beta (ER-β) and proliferating cell nuclear antigen (PCNA) in uterus (A) and vagina (B) of immature mice treated with vehicle (Control), genistein (Gen) or low (8PG-L) or high (8PG-H) dose of 8-prenylgenistein for 7 days. Values were expressed as means ± SEM, *n* = 10. *p < 0.05, **p < 0.01, ***p < 0.001, vs. Control group.

Protein expression of ER-α in uterus (Figure 2(A), p < 0.01) and vagina (Figure 2(B), p < 0.05) was dramatically elevated in genistein- and high dose of 8PG-treated immature mice in comparison with those of immature mice without drug treatment. The similar changes in protein expression of PCNA in uterus and vagina were found in these two groups (*p* < 0.01). Treatment with genistein or low dose of 8PG decreased (*p* < 0.01) protein expression of ER-β in uterus of immature mice.

## Discussion

It is well recognized that naturally occurring phytoestrogens have fewer side effects compared with synthetic oestrogen (Xiao et al. 2015). Seeking effective phytoestrogen is an important and urgent issue in the prevention and treatment of postmenopausal syndrome. In the present study, we studied and compared the estrogenic effects of soy isoflavone genistein and its prenylated form 8PG on uterus and vagina as well as elucidated their regulation on ERs in immature female mice.

The results showed that genistein displayed oestrogenic activity in immature mice as demonstrated by advanced and prolonged oestrous stage and increased tissue index of uterus and vagina in immature mice. Moreover, treatment with genistein stimulated the development and endometrial proliferation of the uterus and the vagina and up-regulated the protein expression of proliferating cell nuclear antigen (PCNA), one proliferation marker. These results were in agreement with studies where the uterus weight was increased in ovary-intact middle-aged rats (Jarić et al. 2018) and ovariectomized mice (Ishimi et al. 2000; Zhang et al. 2018) upon to administration with genistein, indicating a possible action of genistein as endocrine disrupters in reproductive tissues.

8PG, a prenylated form of genistein, did not induce the pathological changes of uterus and vagina when applied with the same dose with genistein in this study, suggesting the chemical modification with side chain of prenylation could weaken the hypertrophic effects of genistein on uterus and vagina, while, 8PG showed beneficial effects on improving bone health in estrogen-deficient mice (Zhang et al. 2018). Additionally, *in vitro* study revealed that 8PG could elevate the osteoblastic function in comparison with that of genistein (Zhang et al. 2008). Therefore, the prenylation on chemical structure of genistein resulted in the increase in therapeutic efficacy on skeletal system and the decrease in side effects on reproductive tissues. Importantly, with the increase in the dosage of 8PG, treatment of immature mice with 8PG (150 mg/kg) led to the hypertrophy of uterus and vagina, fully indicating that 8PG might be a potential candidate as phytoestrogen with tissue-selective activity under a limited dose range and the undesired effects on reproductive tissues may occur with an elevated dose.

Under physiological conditions, the growth and development of uterus and vagina are dependent on distribution and expression of ERs (Xu et al. 2015). Thus, when further exploring the modulation of 8PG on ERs, the protein expression of ER-α and ER-β was determined. Intriguingly, the same as with genistein, high dose of 8PG could enhance protein expression of ER-α, followed by the rise in PCNA protein expression, in uterus and vagina of immature mice. However, neither genistein nor 8PG stimulated the expression of ER-β in uterus and vagina of immature mice. Of note, genistein raised the ER-α expression in uterus, vagina and bone (Zhang et al. 2018), suggesting that ER-α-involved osteopreserve effect of genistein simultaneously triggered the oestrogenic side effects on reproductive system. 8PG down-regulated the expression of ER-α in uterus of ovariectomized mice (Zhang et al. 2018) and dose-dependently up-regulated its expression in uterus and vagina of immature female mice in this study, indicating that the *in vivo* circulating level of oestrogen might interfere with the actions of 8PG on the reproductive system.

In conclusion, 8PG displayed oestrogenic effects on uterus and vagina of immature female mice in this study. The respective contribution of ER subtypes to the oestrogenic actions of 8PG in different tissues should be further explored. In addition, further studies need to be performed to investigate if 8PG could be a candidate as selective oestrogen receptor modulator.
